# Perturbation Training in Anterior Cruciate Ligament Graft Tear and Posterolateral Corner Injury: A Case Report of a Combined Injury

**DOI:** 10.7759/cureus.68750

**Published:** 2024-09-05

**Authors:** Ashish Keoliya, Swapnil U Ramteke

**Affiliations:** 1 Sports Physiotherapy, Ravi Nair Physiotherapy College, Datta Meghe Institute of Higher Education and Research, Wardha, IND

**Keywords:** anterior cruciate ligament (acl) reconstruction, graft failure, perturbation-based balance, posterior corner injury, sports physiotherapy

## Abstract

This case study illustrates how rehabilitation for combination injuries necessitates a thorough, customized strategy that includes perturbation training to address complicated biomechanical impairments because of the complex relationship between the anterior cruciate ligament (ACL) and the posterolateral corner (PCL). An 18-year-old female basketball player visited the Sports Rehab Outpatient Department with a complaint of pain in the posterior aspect of her knee, difficulty fully flexing her right knee joint, and intermittent instability one month after an injury. Her grade 2 ACL tear was accompanied by thinning of the lateral collateral ligament and strain of the popliteofibular ligament as per the MRI findings before the ACL reconstruction surgery. She underwent a knee graft sprain and ACL re-injury. The decision was made to manage the injury conservatively by implementing a rehabilitation program focused on perturbations to improve neuromuscular control and functional stability of the knee. This case report highlights the significance of a multidisciplinary approach, evidence-based interventions (such as the Lysholm score, International Knee Documentation Committee-Subjective Knee Form, and Tempa Scale of Kinesiophobia as outcome measures), and patient-centered care. This study intends to make a significant contribution to sports medicine and orthopedic rehabilitation by clarifying the complexities of rehabilitation in such complicated circumstances.

## Introduction

Although there are well-documented treatment guidelines available for isolated anterior cruciate ligament (ACL) injuries and their rehabilitation, successful rehabilitation becomes more challenging and complicated when additional ligaments are involved. This is particularly evident with the involvement of injuries to the posterolateral corner (PLC), which frequently go undetected and greatly raise the likelihood of ACL reconstruction failure [1]. Knee injuries have more than just physical effects. Sustaining them can have negative social, financial, and even psychological effects on athletes.

Interestingly, with 60% of cases involving significant sports-related treatments on teenagers, knee injuries rank among the most common [2,3]. At least half of all knee injuries involve damage to the ACL; therefore, treating it is essential, but effective treatment frequently necessitates surgery and intense long-term rehabilitation [4]. Even though surgical knee stability restoration, appropriate physical function, advanced postoperative rehabilitation, and advanced age all have a documented role in reducing the risk of re-injury following anterior cruciate ligament reconstruction (ACLR), it is unclear how these factors specifically affect return to sports (RTS) [5].

ACLR primarily affects young, active individuals engaged in competitive sports. Determining the ideal timing for their RTS after the surgery is a frequent concern, but consensus remains elusive. Though the average return time ranges from six months to a year, earlier returns, especially for younger athletes, increase the risk of the ACL tearing again. Age and timing are not the only factors affecting the risk of re-injury. Strength, movement control, and patient-reported outcomes are often evaluated to assess readiness for RTS. Despite numerous proposed testing protocols, there is no standardized approach.

Additionally, passing an RTS test does not guarantee a safe return or prevention of re-injury. The subjective nature and variability of dynamic tests further complicate the matter, as performance requirements vary across different sports [6]. According to statistics, one in five people re-injure one or both knees following ACLR, with men being more susceptible. The rate of re-injury is the highest among males under the age of 18, much higher than that of girls in the same age group [7]. Preventing ACL re-injuries after ACLR requires an understanding of the frequency and severity of this problem as well as the identification of factors that predispose athletes to it [8,9].

Damage to the PLC is commonly caused by intense physical trauma, whether through direct impact or indirect forces. Isolated posterolateral rotatory instability (PLRI) is uncommon but often accompanies injuries to the ACL along with PCL. These combined injuries lead to significant functional impairment, with pain, instability, and progressive deterioration of the joint cartilage if not properly addressed [10]. Although there is an increase in awareness of ACL-PLC injuries, treatment outcomes for ACL-PLC injuries are still elusive. Comparative research, and, more specifically, comparisons of ACL-PLC injuries with isolated ACL injuries, are seriously lacking. A clearer perspective on the recovery and management of these complex injuries needs to be investigated and analyzed [11]. PLC injuries, if left untreated, can be the hidden culprit behind ACLR failure. They frequently occur alongside ACL tears. The PLC comprises three primary ligaments: the popliteofibular ligament (PFL), the popliteus tendon (PLT), and the fibular collateral ligament (FCL). These structures serve as the main stabilizers of the knee, with the FCL providing the strongest protection against leg outward bending. Starting at the front and attaching to the shinbone, the PLT serves as a diagonal support strap. Ultimately, the PFL splits off from the PLT to form a fork with two segments that anchor the fibula’s back to offer further support [12].

Biomechanically, posterolateral corner structures provide primary defense against varus forces and posterolateral tibial rotation around the knee. Additionally, by preventing tibial translation in both directions, they serve a vital secondary function in stabilizing cruciate-deficient knees [13,14]. PLC injuries can be difficult to diagnose because of pain and edema. Successful knee repairs depend on the development of innovative surgical techniques and a deep understanding of the PLC [15,16]. An expert panel’s consensus statement on the diagnosis, classification, treatment, and rehabilitation of PLC injuries reached a high level of agreement (81%), signifying the importance of the topic and identifying areas requiring additional investigation, such as treatment modalities and rehabilitation schedules [17].

This case study illustrates how rehabilitation for combination injuries necessitates a thorough, customized strategy that includes perturbation training to address complicated biomechanical impairments because of the complex relationship between ACL and PLC. To achieve the best outcomes, this case study examines a patient’s rehabilitation journey following a PLC injury and an ACL re-injury, highlighting the significance of a multidisciplinary approach, evidence-based interventions (such as the Lysholm score, International Knee Documentation Committee-Subjective Knee Evaluation Form (IKDC-SKF), and Tempa Scale of Kinesiophobia as outcome measures), and patient-centered care. This study intends to make a significant contribution to sports medicine and orthopedic rehabilitation by clarifying the complexities of rehabilitation in such complicated circumstances.

## Case presentation

Clinical findings

An 18-year-old female basketball player visited the Sports Rehab Outpatient Department with a complaint of pain at the posterior aspect of her knee, difficulty fully flexing her right knee joint, and intermittent instability one month after an injury. She had a history of right knee ACLR surgery six months before the current injury. Her ACL tear was accompanied by a grade 2 tear of the lateral collateral ligament and strain of the PFL as per the MRI findings before the ACLR surgery. In discussing her current injury, she provided a history of it happening during a basketball practice session. The current injury occurred during a basketball practice session, resulting from an awkward landing after a jump, leading to right knee instability and immediate pain. She was unable to walk at that time. For this, she visited an orthopedic surgeon, who advised her to wear a long knee brace along with some analgesic medication and start rehabilitation after a month.

An initial assessment was done after the patient had given informed consent. The patient exhibited slight antalgic gait due to pain and instability, but no significant postural abnormality was observed. Upon physical examination, pain was present around the lateral joint line, along with mild swelling. Varus stress testing showed increased laxity. The dial test was positive at 30°. The range of motion (ROM) testing showed limitations in flexion of the affected side due to discomfort. Active knee flexion was 95° and passively was 100° with an empty end feel. Manual muscle testing was done on the left side and the right side. A conservative physical therapy program was created to target active muscular control and neuromuscular coordination, aiming to compensate for the functional limitations caused by a damaged lateral collateral ligament. Current MRI reports were suggestive of thinning of the lateral collateral ligament with torn PFL and partial ACL graft tear, which confirmed our clinical assessment. The ROM assessment is presented in Table 1.

**Table 1:** Range of motion.

Movement	Right pretreatment	Right posttreatment	Left pretreatment	Left posttreatment
	Active	Passive	Active	Passive
Hip flexion with knee extension	0–80°	0–85°	0–85°	0–90°
Hip extension	0–25°	0–30°	0–30°	0–30°
Hip abduction	0–40°	0–45°	0–45°	0–50°
Hip adduction	0–20°	0–25°	0–25°	0–30°
Hip internal rotation	NA	NA	0–45°	0–45°
Hip external rotation	NA	NA	0–50°	0–50°
Knee flexion	0–95°	0–100°	0–120°	0–135°
Knee extension	95°–0	100°–0	120°–0	135°–0
Ankle plantarflexion	0–50°	0–50°	0–55°	0–55°
Ankle dorsiflexion	0–20°	0–20°	0–20°	0–20°
Ankle inversion	0–30°	0–30°	0–35°	0–35°
Ankle eversion	0–15°	0–15°	0–20°	0–20°

The patient noted that her knee condition significantly affected her ability to play basketball and perform daily activities. She was eager to return to her sport and was highly motivated to adhere to a rehabilitation program. The decision was made to manage the injury conservatively with a rehabilitation program focused on perturbation training, aiming to improve neuromuscular control and functional stability of the knee. The initial goals of the rehabilitation were to reduce pain and swelling, restore ROM, and begin neuromuscular training to stabilize the knee joint.

Radiological findings

**Figure 1:**
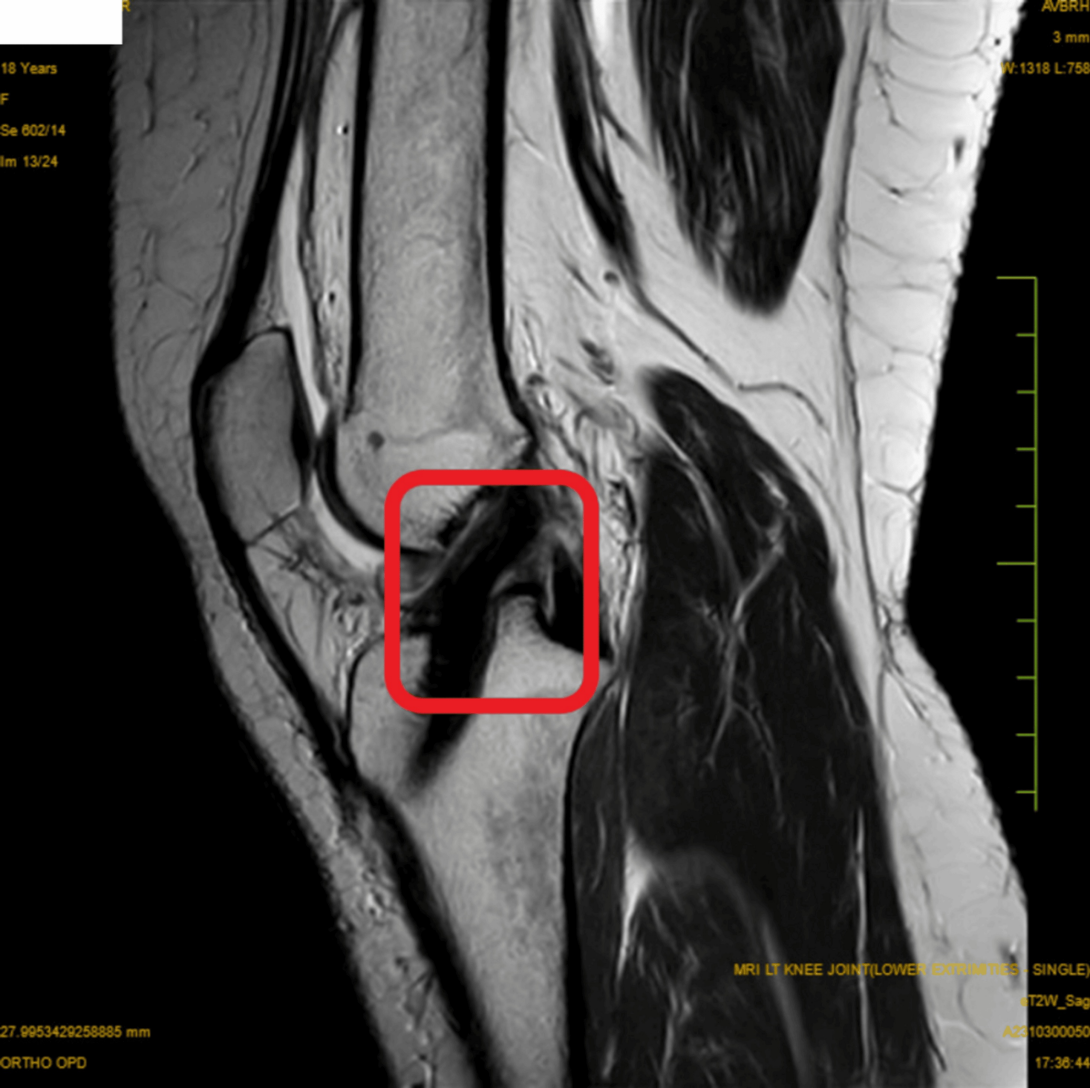
MRI showing the partial anterior cruciate ligament graft tear.

The radiological examination is shown in Figure 1.

Physiotherapy management

The International Classification of Functioning, Disability and Health was taken into consideration when developing the rehabilitation program, as shown in Table 2.

**Table 2:** Physiotherapy rehabilitation program.

Phase	Time frame	Goals	Exercises	Frequency
Phase 1: Acute phase	0–4 weeks	Reduce pain and swelling. Protect the injured graft. Maintain the range of motion and strength in unaffected areas	Rest and ice. Elevation and compression. Gentle range of motion exercises. Isometric quadriceps exercises. Ankle pumps. Partial weight-bearing with crutches with long knee brace	3–4 times daily (ice and elevation), 2–3 times daily (exercises)
Phase 2: Early rehabilitation	4–8 weeks	Gradually restore range of motion. Begin weight-bearing exercises. Initiate neuromuscular control training	Range of motion exercises (heel slides, passive knee extension). Straight leg raises. Balance exercises on a stable surface	1–2 times daily, weight-bearing exercises 2–3 times daily
Phase 3: Intermediate rehabilitation	8–12 weeks	Improve strength and endurance. Enhance neuromuscular control. Increase weight-bearing activities	Progressive resistance exercises (leg press, hamstring curls). Stationary cycling. More focus on perturbation training on stable and unstable surfaces (e.g., tilt boards, foam pads). Single-leg balance exercises	3–4 times weekly (resistance and cycling). Daily (perturbation and balance)
Phase 4: Advanced rehabilitation	12–20 weeks	Increase power and agility. Prepare for return to sport-specific activities	Plyometric exercises (jumping drills, box jumps). Agility drills (ladder drills, cone drills). Sport-specific drills (dribbling, shooting). Advanced perturbation training (dynamic balance and reaction exercises)	3–4 times weekly (plyometrics and agility). Daily (sport-specific and perturbation)
Phase 5: Return to sport	20+ weeks	Achieve full functional recovery. Ensure psychological readiness for return to sport	Continue advanced strengthening and conditioning. Full sport-specific training. Gradual return to competitive play. Ongoing perturbation training to maintain neuromuscular control	3–4 times weekly (strength and conditioning). Daily (sport-specific and perturbation)

Outcome measures

**Figure 2:**
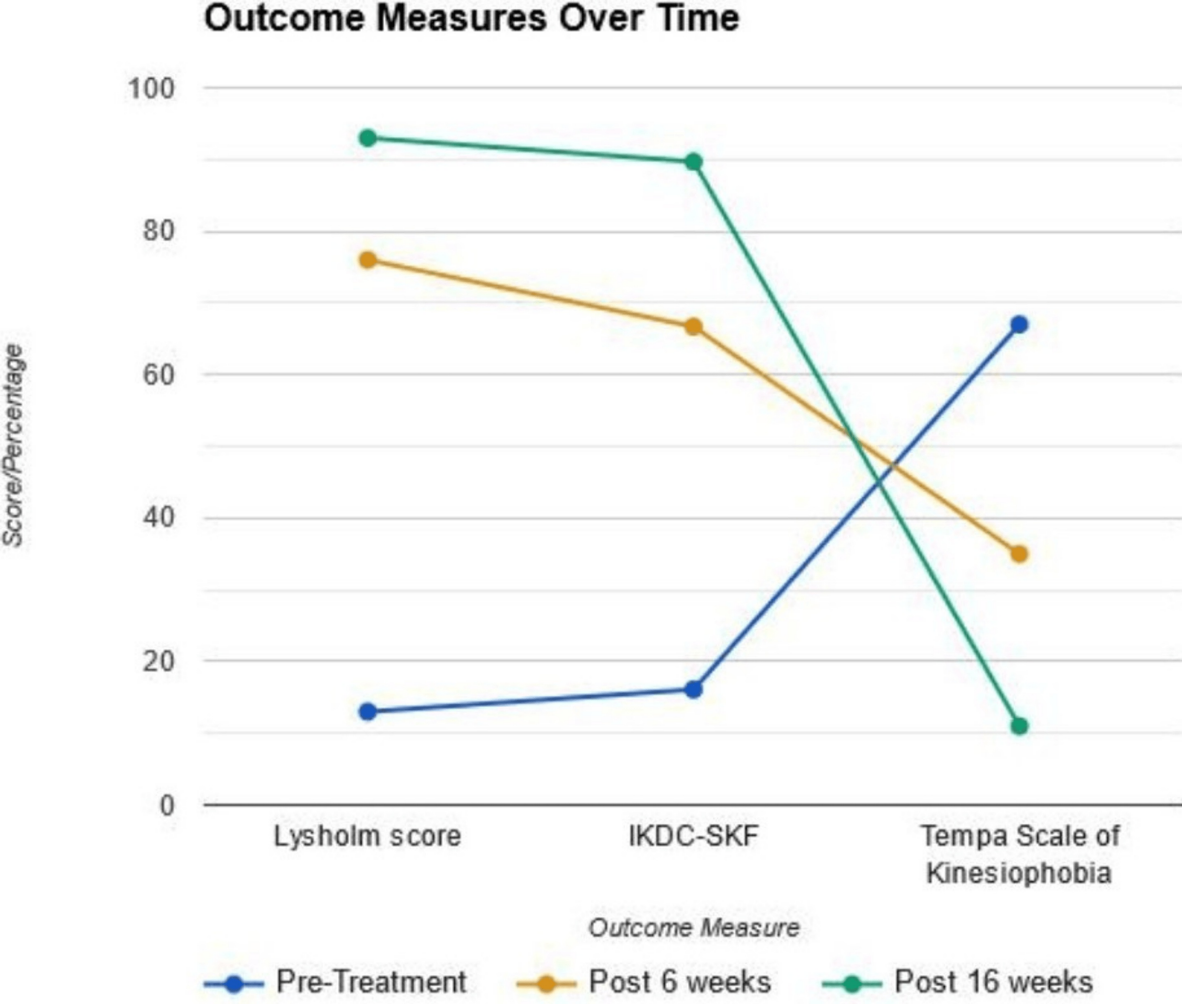
Graphical representation of pre and post-outcome measures. IKDC-SKF = International Knee Documentation Committee-Subjective Knee Evaluation Form

Analysis of the data from outcome measures throughout the treatment period revealed significant improvements in patient function and reduced fear of movement. A different therapist assessed the outcome measures. The outcome measures were recorded at baseline and the conclusion of the 16-week intervention. Knee function scores, measured by both the Lysholm score (assessing activities of daily living) and the IKDC-SKF score (focusing on sports activities), displayed a dramatic increase. The Lysholm score increased from 13% pretreatment to a remarkable 93% after 16 weeks, while the IKDC-SKF score showed a similar trajectory, rising from 16.1% to 89.7% over the same timeframe. This translates to a substantial improvement in the patient’s ability to perform daily activities and participate in sports. Furthermore, the Tempa Scale of Kinesiophobia, which measures fear of movement, demonstrated a significant decrease. The patient entered treatment with a score of 67, indicating severe fear of movement. This score dropped to a reassuring 11 by the end of the treatment period, signifying a substantial reduction in kinesiophobia and a transition from a state of severe fear to minimal or no fear of movement. A graphical representation of pre- and post-outcome measures is shown in Figure 2 and a tabular presentation is in Table 3.

**Table 3:** Pre and post-outcome measures. Tempa Scale of Kinesiophobia: 17 = no kinesiophobia, >37 = kinesiophobia, 68 = severe kinesiophobia. IKDC-SKF = International Knee Documentation Committee-Subjective Knee Evaluation Form

Outcome measures	Pre-treatment	After 6 weeks	After 16 weeks
Lysholm score	13%	76%	93%
IKDC-SKF	16.1%	66.7%	89.7%
Tempa Scale of Kinesiophobia	67	35	11

## Discussion

This case report provides an excellent example where ACL graft strain, in combination with injury to the PLC, highlights that a perfect rehabilitation program can be challenging but paramount. In this patient, therefore, the dual goals of achieving knee strength and stability to get back into the workforce and protecting the healing graft made a carefully designed training regimen a must.

Previous research by Dean et al. has emphasized the importance of concurrently managing both ACL and suspected PCL injuries [18]. Their review highlights the critical role of understanding local anatomy, employing accurate diagnostics, and utilizing appropriate surgical techniques to achieve ideal clinical outcomes. In this case, the partially torn PFL within the PLC was not addressed during the initial ACL repair surgery, likely contributing to further instability and ultimately leading to graft rupture. This emphasizes the crucial role of identifying and managing PLC injuries alongside ACLR whenever suspected.

Neuromuscular and accompanying strength deficits post-ACL surgeries can persist for years [19]. Perturbation training, a key element in this patient’s rehabilitation program, has been shown to improve knee stability and reduce feelings of instability [20]. This is particularly relevant for female patients, who tend to activate their quadriceps more than their hamstrings during functional activities compared to men [21]. This quadriceps dominance can place greater stress on the ACL, increasing the risk of injury. Perturbation training, by promoting balanced muscle activation, can be a valuable tool in ACL rehabilitation programs, especially for female patients. Perturbation training improves knee stability by activating the neuromuscular system. This effect is achieved through improvements in proprioception, neuromuscular control, kinesthesia, and dynamic joint stability. The results of our case report align with the findings of Wilk et al. regarding the effectiveness of perturbation training in rehabilitation [22].

As a single-case study, the generalizability of the findings may be limited. It is a major limitation of the study. Additionally, the decision not to address the PLC injury during the initial surgery raises questions about the surgical strategy and the potential impact of preoperative imaging on identifying the extent of the PLC pathology. Furthermore, the lack of extensive long-term follow-up limits our understanding of the durability of the rehabilitation program and the patient’s functional recovery over time.

This case report underscores the importance of a comprehensive evaluation and treatment approach for injuries involving both the ACL and PLC. The findings suggest that neglecting to address PLC injuries during ACLR can significantly compromise the surgical outcome and increase the risk of re-injury. Healthcare professionals should prioritize identifying and managing PLC pathology alongside ACLR, especially when suspected. This case also highlights the potential benefits of perturbation training in enhancing knee stability and reducing the risk of re-injury, particularly for female patients with quadriceps dominance.

## Conclusions

This case study emphasizes how crucial it is to take the patient’s age, gender, and level of injury complexity into account when creating a rehabilitation plan. The effective recovery of a young athlete with a complex knee injury serves as an example of this concept. The improvements are based on subjective reports from the patient, which can introduce bias. Objective, standardized measures of knee function and stability would provide stronger evidence for the effectiveness of the rehabilitation program.
